# *Carnobacterium:* positive and negative effects in the environment and in foods

**DOI:** 10.1111/j.1574-6976.2007.00080.x

**Published:** 2007-08

**Authors:** Jørgen J Leisner, Birgit Groth Laursen, Hervé Prévost, Djamel Drider, Paw Dalgaard

**Affiliations:** 1 Department of Veterinary Pathobiology, Faculty of Life Sciences, University of Copenhagen Copenhagen, Denmark; 2 Department of Seafood Research, Danish Institute for Fisheries Research, Technical University of Denmark Lyngby, Denmark; 3 UMR INRA-1014 SECALIM, Ecole Nationale d'Ingénieurs des Techniques des Industries Agricoles et Alimentaires (ENITIAA) Nantes, France

**Keywords:** *Carnobacterium*, natural environment, antimicrobial properties, food spoilage, food safety, probiotics

## Abstract

The genus *Carnobacterium* contains nine species, but only *C. divergens* and *C. maltaromaticum* are frequently isolated from natural environments and foods. They are tolerant to freezing/thawing and high pressure and able to grow at low temperatures, anaerobically and with increased CO_2_ concentrations. They metabolize arginine and various carbohydrates, including chitin, and this may improve their survival in the environment. *Carnobacterium divergens* and *C. maltaromaticum* have been extensively studied as protective cultures in order to inhibit growth of *Listeria monocytogenes* in fish and meat products. Several carnobacterial bacteriocins are known, and parameters that affect their production have been described. Currently, however, no isolates are commercially applied as protective cultures. Carnobacteria can spoil chilled foods, but spoilage activity shows intraspecies and interspecies variation. The responsible spoilage metabolites are not well characterized, but branched alcohols and aldehydes play a partial role. Their production of tyramine in foods is critical for susceptible individuals, but carnobacteria are not otherwise human pathogens. *Carnobacterium maltaromaticum* can be a fish pathogen, although carnobacteria are also suggested as probiotic cultures for use in aquaculture. Representative genome sequences are not yet available, but would be valuable to answer questions associated with fundamental and applied aspects of this important genus.

## Introduction

Carnobacteria are ubiquitous lactic acid bacteria (LAB) isolated from cold and temperate environments. More importantly from a practical viewpoint, they also frequently predominate in a range of foods, including fish, meat, and some dairy products. In this regard, they have been extensively studied in the last two decades as protective cultures, to inhibit pathogenic and spoilage microorganisms, and as potential spoilage bacteria in chilled seafood and meat products. In addition, owing to their presence in the aqueous environment, their importance as fish pathogens or probiotic cultures in the aquaculture industry has been examined.

The genus *Carnobacterium* currently consists of nine species, but only two of these, *Carnobacterium divergens* and *C. maltaromaticum* (formerly *C. piscicola*) are frequently encountered in the environment and in foods (Table 1). This review concerns the importance of these two species in dairy, meat and fish products and in the aquatic environment. The taxonomy and methods for isolation and identification of carnobacteria are not described. It is, however, important to note that older studies frequently used carbohydrate fermentation patterns to distinguish between *C. divergens* and *C. maltaromaticum*. These methods are now considered less reliable than molecular methods, and results relying on phenotypic criteria must be interpreted with caution (Hammes & Hertel, 2003; Laursen *et al.*, 2005).

**Table 1:** Compilation of *Carnobacterium* species and known sources*

		Isolated from	
		
Species	Isolation frequency	Food products	Environment
*C. alterfunditum*	Very low	Not reported	Live fish, polar lakes/sea, deep sea sediment
*C. divergens*	High	Dairy, meat, fish, shrimp	Intestine of live fish, *Sphagnum* pond (U)*, alpine permafrost (U)
*C. funditum*	Very low	Not reported	Polar lakes/sea, intestine of live fish, marine sponge
*C. gallinarum*	Low	Meat	Live fish
*C. inhibens*	Very low	Not reported	Atlantic salmon
*C. maltaromaticum*†	High	Dairy, meat, fish, shrimp	Live/diseased fish, moth larval midgut, polar sea, deep sea (U), cold and alkaline tufa columns, Japanese lakes (U), *Sphagnum* pond (U)
*C. mobile*	Low	Meat, shrimp	Live fish
*C. pleistocenium*	Very low	Not reported	Permafrost tunnel
*C. viridans*	Very low	Meat	Not reported
*Carnobacterium* spp.‡	NA	See‡	Andean wetlands (U), Canadian oil sands tailing pond (U), cow rumen (U), deep sea (U), polar areas, pufferfish organs (U), spent mushroom compost, pig effluent-impacted environment (U), watershed polluted with horse manure

* References are quoted in the text except for unpublished (U) studies that include the following accession numbers (EF090688; AM269906; AB248932; AB248935; AB260993; AM711880; EF420230; AY244979; AM111051; DQ778093; DQ337521; DQ337531). In some cases (EF090688, AM269906, AB260993), the source of the sequence was listed as *Carnobacterium* sp. However, comparison of the 16S rRNA gene sequences with additional carnobacterial 16S rRNA gene sequences in the databases (http://www.ncbi.nlm.nih.gov) allowed us to allocate these sequences to specific carnobacterial species.

† Formerly *C. pisciciola* Mora *et al.* (2003). Two human clinical isolates of this species are known, Chmelař*et al.* (2002), Xu *et al.* (1997); and AF113133. Two clinical isolates of *C. divergens* have also been described (AY650920).

‡ Examples of habitats described for cultured and noncultured samples. Sequences for samples obtained from foods are, with a few exceptions [e.g. milk (EF204312)], allocated to known species.

NA, not applicable.

## Distribution in the natural environment and foods

### Natural environment

Among the nine reported species of *Carnobacterium*, only two species, *C. divergens* and *C. maltaromaticum*, are frequently isolated from various sources (Table 1). Four species, *C. alterfunditum*, *C. funditum*, *C. gallinarum* and *C. mobile*, have been isolated a few times from at most three sources (Table 1). Three species, *C. inhibens*, *C. pleistocenium* and *C. viridans*, have only been isolated from one source (Jöborn *et al.*, 1999; Holley *et al.*, 2002; Pikuta *et al.*, 2005), but it should be noted that *Carnobacterium* spp. related to, for example, *C. inhibens* have been described (Simpson *et al.*, 2004). *Carnobacterium* spp. appear to have both the temperate and polar aquatic environments as habitats including live fish (see also ‘Carnobacteria as pathogenic organisms and/or probiotic cultures’), marine sponges (Li & Liu, 2006), Antarctic lakes (Franzmann *et al.*, 1991; Bratina *et al.*, 1998), Arctic and Antarctic sea water as well as the deep sea (Galkin *et al.*, 1999; Groudieva *et al.*, 2004; Newberry *et al.*, 2004; Toffin *et al.*, 2004; Lauro *et al.*, 2007), aquous alkaline tufa columns from Greenland (Schmidt *et al.*, 2006), and freshwater habitats from the temperate clima zone, including a *Sphagnum* pond (Leisner *et al.*, unpublished results) and rivers in the northwest region of Spain (González *et al.*, 1999) (Table 1). Although *C. maltaromaticum* and/or *C. divergens* have been isolated from tropical fish products, including smoked surubim, a Brazilian tropical freshwater fish (Alves *et al.*, 2005), and from vacuum-packed tuna caught in the Indian Ocean and processed in Sri Lanka (Emborg *et al.*, 2005), it cannot, at least in the latter case, be excluded that this is due to contamination associated with repackaging in Denmark.

The presence of carnobacteria has also been demonstrated in the terrestrial environment, including a field treated with whey (Coombs & Brenchley, 1999), Canadian winter soil (Walker *et al.*, 2006), permafrost ice (Pikuta *et al.*, 2005; Katayama *et al.*, 2007), a compost pile (Ntougias *et al.*, 2004), a collapsed horse manure pile (Simpson *et al.*, 2004), the larval midgut of a moth species (Shannon *et al.*, 2001), and other sources (Table 1). It appears that the temperate/polar aquatic and terrestrial environments are both natural habitats.

*Carnobacterium divergens* and *C. maltaromaticum* possess traits that may play a role in their survival in these surroundings. One study has reported that a *Carnobacterium* sp. soil isolate related to *C. maltaromaticum* survived 48 serial freeze–thaw cycles better than *Escherichia coli* and an *Enterococcus* sp. soil isolate, but worse than a few other soil isolates, including an *Acinetobacter* sp. (Walker *et al.*, 2006). Indeed, these organisms may survive freezing for considerable periods of time, as witnessed by the isolation of a *Carnobacterium* sp. preserved in a permafrost ice wedge for 25 000 years (Katayama *et al.*, 2007). Additional data on the abilities of carnobacteria to grow at low temperatures and to survive frozen storage is available for food products. Also, a cold-active β-galactosidase from *C. maltaromaticum* and a cold-adapted alanine dehydrogenase from a *Carnobacterium* sp. related to *C. alterfunditum* have been reported (Coombs & Brenchley, 1999; Galkin *et al.*, 1999). Some carnobacterial isolates originate from natural high-pressure habitats (Lauro *et al.*, 2007), and additional data on resistance to pressure have been reported for high-pressure-processed foods. It has not been described how other physical/chemical parameters such as salt content, atmosphere and pH affect survival and growth of these organisms in the natural environment.

With respect to energy-yielding substrates, one species, *C. maltaromaticum*, expresses chitinase activity (Leisner & Ingmer, unpublished data). This may facilitate adherence to and survival on zooplankton as suggested for enterococci (Signoretto *et al.*, 2005), and also explain the isolation of this species from the midgut of the larval stage of a species of moth (Shannon *et al.*, 2001). Interestingly, the arginine deiminase pathway is expressed by the most frequently encountered species, *C. divergens*, *C. gallinarum*, *C. maltaromaticum* and *C. mobile* (e.g. Collins *et al.*, 1987, 2002; Leisner *et al.*, 1994b; Schillinger & Holzapfel, 1995) but not by *C. inhibens* or *C. viridans* (Jöborn *et al.*, 1999; Collins *et al.*, 2002; Holley *et al.*, 2002). Arginine is not a substrate that results in growth of *C. alterfunditum* and *C. funditum* (Franzmann *et al.*, 1991), and information is not available for *C. pleistocenium* (Pikuta *et al.*, 2005). This amino acid may represent an additional energy source for growth and survival when carbohydrates are scarce, and it may offer protection against acid stress, as described for *Enterococcus faecalis* and related species (Marquis *et al.*, 1987). Finally, carnobacteria are able to catabolize a range of carbohydrates, although there are considerable interspecies and intraspecies heterogeneities. Examples of sources of such carbohydrates include animals (e.g. chitin and, to some extent, lactose, although the targets of the lactose hydrolytic activity shown by carnobacteria may instead be byproducts of plant sugar polymers and saccharides adsorbed to humic acid substances in the soil) (Coombs & Brenchley, 2001), plants (e.g. salicin and sucrose), fungi (e.g. chitin and trehalose), prokaryotes (*N*-acetylglucosamine), and living organisms in general (ribose) (Shaw & Harding, 1984; Lai & Manchester, 2000; Rudi *et al.*, 2004). The importance of these abilities for growth and survival in the environment deserves further study. The genome sizes of *C. alterfunditum*, *C. divergens* and *C. pleistocenium* strains and a *Carnobacterium* sp. AT7 deep sea isolate have been estimated to be 2.9, 3.2, 3.2 and 2.4 Mb, respectively (Daniel, 1995; Pikuta *et al.*, 2005), and for the AT7 isolate, the data are reported in a database described by Liolios (2006). These sizes are relatively large in comparison to many other LAB, suggesting that carnobacterial genomes may encode a range of genes that makes them well adapted to deal with environmental challenges.

Even if carnobacteria are well adapted to temperate or polar aqueous environments, this does not always provide improved ability to survive as compared to other bacteria. Thus, the actual abilities of strains of *C. divergens* and *C. maltaromaticum* isolated from a *Sphagnum* pond to survive in water from this source were not improved as compared to related gram-positive bacteria originating from other sources (Leisner *et al.*, unpublished data). At present, therefore, the precise mechanisms by which *Carnobacterium* spp. persist in the natural environment and their underlying genetics are not known. Finally, it should be noted that the environments from which carnobacteria can be isolated are not always as extreme as they appear at first sight. Thus, *Carnobacterium* spp. isolated from Lake Vanda, Antarctica were found at a depth of 61 m, where the temperature was *c*. 15–20°C (Bratina *et al.*, 1998).

### Dairy, fish and meat products

High concentrations of bacteria (>10^6^–10^7^ CFU g^−1^) in food are typically required before their activity is sufficient to influence the sensory properties of a product. For this to happen, occurrence and growth kinetics are the key parameters, and databases, including ComBase, are available to determine the effect of food storage conditions and product characteristics on growth of specific bacteria (McMeekin *et al.*, 2006). However, information on carnobacteria has not yet been included in such databases.

*Carnobacterium maltaromaticum* and another *Carnobacterium* sp. have been isolated from milk (Miller *et al.*, 1974; EF204312), and they, in addition to *C. divergens*, have been detected in soft cheeses, including mold-ripened brie (Millière *et al.*, 1994), mozzarella (Morea *et al.*, 1999), Camembert (Cailliez-Grimal *et al.*, 2005) and other types (Millière & Lefebvre, 1994, Cailliez-Grimal *et al.*, 2007). The high concentration of *C. divergens* in the curd of mozzarella, exposed to 10–37°C during processing, is in agreement with the maximum growth temperature (40°C) of this species (Collins *et al.*, 1987).

The occurrence of *Carnobacterium* in dairy products (and other foods) is most likely underreported. This is due to the common use of acetate containing media, particularly MRS agar (Oxoid CM361) or Rogosa agar (Oxoid CM0627), for enumeration of LAB (de Man *et al.*, 1960). Growth of *Carnobacterium* is inhibited by acetate, and both MRS and Rogosa agar media significantly underestimate concentrations in food (Leblanc *et al.*, 1997; Sakala *et al.*, 2002; Hammes & Hertel, 2003; Susiluoto *et al.*, 2003; Chenoll *et al.*, 2007).

*Carnobacterium divergens* and *C. maltaromaticum* are present in seafood and are able to grow to high concentrations in different fresh and lightly preserved products. Studies of naturally contaminated products suggest which storage conditions and product characteristics select for carnobacteria as compared to the other bacteria present in seafood. For chilled fresh seafood, we have found no reports where *C. divergens* and *C. maltaromaticum* dominated the microbial communities in aerobically stored products, but this has been reported for modified atmosphere-packed (MAP) coalfish, cod, pollack, rainbow trout, salmon, shrimp, swordfish and tuna (Mauguin & Novel, 1994; Leblanc *et al.*, 1997; Emborg *et al.*, 2002, 2005; Franzetti *et al.*, 2003; Rudi *et al.*, 2004). After frozen storage (−20 to −30°C for 5–8 weeks), *C. divergens* and *C. maltaromaticum* seem to be particularly prominent in chilled MAP fish, as has been reported for cod, garfish, salmon, and tuna (Guldager *et al.*, 1998; Emborg *et al.*, 2002, 2005; Dalgaard *et al.*, 2006). In addition to freezing, these carnobacteria are relatively resistant to high-pressure processing and are found in high concentrations in vacuum-packed and chilled squid mantle and cold-smoked salmon previously treated with 200–400 MPa for 15–20 min (Paarup *et al.*, 2002; Lakshmanan & Dalgaard, 2004). In vacuum-packed cold-smoked or sugar-salted (‘gravad’) seafood with 3–7% NaCl in the water phase and a pH of 5.8–6.5, high concentrations of *C. divergens* and *C. maltaromaticum* are particularly common, as reported for halibut, rainbow trout, salmon, surubim, and tuna (Jeppesen & Huss, 1993; Leisner *et al.*, 1994a; Pilet *et al.*, 1995; Leroi *et al.*, 1998; Lyhs *et al.*, 1998, 2002; Paludan-Müller *et al.*, 1998; Truelstrup Hansen & Huss, 1998; Jørgensen *et al.*, 2000; González-Rodríguez *et al.*, 2002; Alves *et al.*, 2005; Emborg & Dalgaard, 2006). High concentrations of *C. maltaromaticum* are also reported in salted lumpfish roe, and it has been isolated from frozen, smoked mussels (Basby *et al.*, 1998; Tahiri *et al.*, 2004). Finally, for cooked seafood, high concentrations of *C. maltaromaticum* have been detected in MAP shrimp after storage at 2–8°C (Mejlholm *et al.*, 2005). Both *C. divergens* and *C. mobile* were isolated from cooked and brined MAP shrimps (with NaCl, benzoic acid, citric acid and sorbic acid) after storage at 2–8°C (Dalgaard *et al.*, 2003; Laursen *et al.*, 2005). Clearly, carnobacteria are common in chilled fresh and lightly preserved seafood, but at higher storage temperatures (15–25°C), other species, including *Enterococcus* spp., more frequently dominate the spoilage microbial community of seafood (Dalgaard *et al.*, 2003).

*Carnobacterium divergens* and *C. maltaromaticum* are able to grow in meat products at temperatures as low as 2 to −1.5°C (McMullen & Stiles, 1993; Sakala *et al.*, 2002; Jones, 2004), and they are frequently predominant members (up to 50%*C. divergens* and up to 26%*C. maltaromaticum* of the gram-positive or LAB isolates obtained) of the microbial community of raw meat (beef, pork, lamb, and poultry). The two species are found irrespective of whether products have been stored aerobically, vacuum packaged, or subjected to modified atmospheres, including gas compositions of CO_2_/N_2_ (%) ranging from 10 : 90 to 80 : 20 (Shaw & Harding, 1984; Grant & Patterson, 1991; McMullen & Stiles, 1993; Barakat *et al.*, 2000; Sakala *et al.*, 2002; Susiluoto *et al.*, 2003; Jones, 2004; Björkroth *et al.*, 2005; Laursen *et al.*, 2005; Vihavainen *et al.*, 2007). One study demonstrated the presence of *C. divergens* in beef stored in air but not in MAP beef with increased concentrations of O_2_ (20–40%) in addition to CO_2_ (40%) (Ercolini *et al.*, 2006). The growth of *C. funditum* is impaired by oxygen (Franzmann *et al.*, 1991), and it will be of interest to study further whether addition of O_2_ to the gas composition of modified atmospheres consistently inhibits growth of carnobacteria, as has been observed for some other bacteria (Emborg *et al.*, 2005).

Further studies are needed in order to determine whether differences in the presence of carnobacteria in meat are due to variations in storage conditions or variations in contamination levels at the processing plants. The source of carnobacteria in meat products is most probably the processing plant, as these organisms have not been isolated from the gastrointestinal system or skin of chicken, cattle, pigs or sheep, except for one unpublished study (cow rumen, AY244979). Thus, the source of contamination of broiler carcasses by *C. divergens* and *C. maltaromaticum* was shown to be the air in the processing plant and not incoming broiler chickens (Vihavainen *et al.*, 2007). In fact, two studies confirmed that *C. divergens* and *C. maltaromaticum* present in the raw material were eliminated or reduced in number by cooking of a processed meat product, ham (Samelis *et al.*, 1998, 2000).

*Carnobacterium divergens* and *C. maltaromaticum* have, however, been detected in a variety of processed meat products, including the cured pork product bacon (Shaw & Harding, 1984), ham (Borch & Molin, 1988; Jack *et al.*, 1996), a Danish processed pork product (‘rullepølse’) (Laursen *et al.*, 2005 and unpublished results), various Spanish processed meat products (Chenoll *et al.*, 2007), cooked poultry meat (Barakat *et al.*, 2000), pressure-treated (408–888 MPa) chicken (O'Brien & Marshall, 1996), and irradiated pork and chicken (Grant & Patterson, 1991). On a few occasions, *C. maltaromaticum* has even been isolated from fermented sausages (Schillinger & Lücke, 1987; Larrouture-Thiveyrat *et al.*, 2003); as noted by Hammes & Hertel (2003), this contrasts with the usual nonaciduric habitats of carnobacteria. Other *Carnobacterium* spp. isolated from processed meat products include *C. gallinarium* (irradiated chicken) (Thornley, 1957; Collins *et al.*, 1987), *C. mobile* (cooked turkey) (Chenoll *et al*,. 2007) and *C. viridans* (vacuum-packed Bologna sausage) (Holley *et al.*, 2002). Although these organisms are frequently isolated from processed meat products, it has been suggested that they are rarely present in high numbers, and that terminal spoilage of cooked, cured meats is primarily caused by aciduric LAB (Samelis, 2006; Chenoll *et al.*, 2007).

Finally, a *Carnobacterium* sp. has also been isolated from egg contents, and it was shown that this isolate had a similar ability to penetrate the eggshell and contaminate the whole egg as various other gram-positive and gram-negative bacteria (De Reu *et al.*, 2006).

Transmission routes for carnobacteria from the natural environment into food-manufacturing plants and further on to dairy, fish and meat products are not known to any extent. Knowledge on this topic is essential to evaluate if species and intraspecific clusters that differ in spoilage ability (as discussed later) also have different colonization abilities. Such knowledge would also illuminate the feasibility of using the production of metabolites, especially tyramine, by *C. divergens* and *C. maltaromaticum* as an index of microbial spoilage of specific fish and meat products (Edwards *et al.*, 1987; Dainty & Mackey, 1992; Leisner *et al.*, 1994a; Dainty, 1996; Jørgensen *et al.*, 2000; Emborg *et al.*, 2002; Laursen *et al.*, 2006).

## Functional properties of carnobacteria

### Bacteriocins and antimicrobial properties

Carnobacteria with the ability to produce antimicrobial peptides, bacteriocins, are commonly encountered in foods (e.g. Lewus *et al.*, 1991; Coventry *et al.*, 1997). The characterized carnobacterial bacteriocins belong to class I and class II [e.g. Drider *et al.* (2006) for bacteriocin nomenclature]. So far, only one class I bacteriocin (lantibiotic), UI149, has been reported to be produced by *Carnobacterium* (Stoffels *et al.*, 1992a, b). In contrast, ten amino acid-sequenced bacteriocins belong to class II, and most of these more specifically to class IIa, which comprises small pediocin-like peptides (Table 2). The inhibition spectrum of carnobacterial class IIa bacteriocins includes *Listeria*, and antimicrobial activity is exerted by pore formation, dissipation of membrane potential, and leakage of internal low molecular weight substances (Suzuki *et al.*, 2005; Drider *et al.*, 2006).

**Table 2:** Bacteriocins produced by carnobacteria

Species	Bacteriocin (class)*	Gene	Unprocessed precursor/ mature chain†	Location of gene	Accession number	References
*Cm*‡	Carnobacteriocin A (IIc)	*cbnA*	71/53	Plasmid	P38578	Worobo *et al.* (1994)
	Piscicolin 61§					Holck *et al.* (1994)
	Carnobacteriocin BM1 (IIa)	*cbnBM1*	61/43	Chromosome	P38579	Quadri *et al.* (1994)
	Carnobacteriocin B1§					
	Piscicocin V1b§					Bhugaloo-Vial *et al.* (1996)
	Carnocin CP51§					Herbin *et al.* (1997)
	Carnobacteriocin B2 (IIa)	*cbnB2*	66/48	Plasmid	P38580	Quadri *et al.* (1994)
						Wang *et al.* (1999)
	Carnocin CP52§					Herbin *et al.* (1997)
	A9b§					Nilsson *et al.* (2002)
	Piscicolin 126 (IIa)	*pisA*	62/44	Chromosome	P80569	Jack *et al.* (1996)
	Piscicocin V1a§					Bhugaloo-Vial *et al.* (1996)
						Gursky *et al.* (2006)
	Piscicocin CS526 (IIa)	ND	X/≥43	ND		Yamazaki *et al.* (2005)
*Cd*	Divercin V41 (IIa)	*dvn41*	66/43	Chromosome	CAA11804	Métivier *et al.* (1998)
	Divergicin M35 (IIa)	ND	X/43	ND	P84962	Tahiri *et al.* (2004)
	Divergicin A (IIc)	*dvnA*	75/46	Plasmid	AAZ29031	Worobo *et al.* (1995)
						van Belkum & Stiles (2006)
	Divergicin 750 (IIc)	*dvn750*	63/34	Plasmid	2209292A	Holck *et al.* (1996)
*Carnobacterium* sp.	Carnocin H (II)	ND	Partial sequence¶	ND		Blom *et al.* (2001)
	Carnocin UI149 (I)	ND	Partial sequence∥	ND	P36960	Stoffels *et al.* (1992b)

* Classification of bacteriocins based on review by Drider *et al.* (2006).

† Numbers of amino acids.

‡ *Carnobacterium divergens (Cd)*, *Carnobacterium maltaromaticum (Cm)*.

§ Synonyms.

¶ Approximately 75 amino acids.

∥ 35–37 amino acids.

ND, not determined.

Class IIa bacteriocins are ribosomally synthesized as inactive prepeptides that are modified by posttranslational cleavage of the N-terminal peptide leader at a double-glycine site in order to release mature and active cationic peptides. Carnobacterial class IIa bacteriocins (Table 2) have similar amino acid sequences, with a YGNGV(X)C(X)_4_C motif (X denotes any amino acid) near the N-terminus of the mature peptide. Two cysteine residues form a disulfide bond in the N-terminal region, and there is an amphipathic α-helix near the C-terminus. The N-terminal region is relatively hydrophilic and conserved, whereas the C-terminus is hydrophobic and diverse. To establish the structure–activity relationships of carnobacterial bacteriocins, the structures of carnobacteriocin B2 and divercin V41 were modified (Quadri *et al.*, 1997a; Bhugaloo-Vial *et al.*, 1999). Thus, amino acid substitutions closer to the N-terminus in carnobacteriocin B2 drastically reduced or eliminated antimicrobial activity, whereas this was not so for substitutions close to the C-terminal part (Quadri *et al.*, 1997a). Divercin V41 contains a second disulfide bond located in the C-terminal region (Métivier *et al.*, 1998). When the C-terminal region of divercin V41 was separated from the N-terminus by endoproteinase Asp-N, only the C-terminal fragment was active. After trypsin cleavage next to lysine at position 42 or disulfide reduction, the C-terminus lost its inhibitory activity. These results suggested that both hydrophobicity and folding imposed by this second disulfide bond were essential for antilisterial activity of the C-terminal hydrophobic peptide (Bhugaloo-Vial *et al.*, 1999). Chemical oxidation of tryptophan residues by *N*-bromosuccinimide showed that these residues were crucial for inhibitory activity, as modification of any one of them rendered divercin V41 inactive (Bhugaloo-Vial *et al.*, 1999). The three-dimensional structure of carnobacteriocin B2, its precursor and the corresponding immunity protein have been solved by nuclear magnetic resonance (Wang *et al.*, 1999; Sprules *et al.*, 2004a, b). These are, at present, the only reported carnobacterial protein structures.

Carnobacterial class II bacteriocins that contain a double-glycine-type leader peptide are transported by a dedicated ATP-binding cassette (ABC) transport system (Drider *et al.*, 2006). In contrast, divergicin A does not require a secretion protein, as the transport depend on the general cellular secretion (sec) pathway (Worobo *et al.*, 1995).

**Fig. 1:**
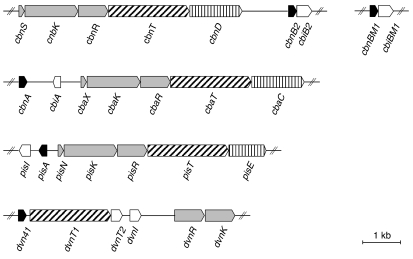
Gene loci involved in carnobacteriocins B2, BM1, A, piscicolin 126 and divercin V41 production and immunity. First line, left: carnobacteriocin B2 locus (plasmid pCP40, accession number L47121). First line, right: carnobacteriocin BM1 chromosomal locus (L29058). Second line: carnobacteriocin A locus (plasmid pCP49, AF207838). Third line: piscicolin 126 chromosomal locus (AF275938). Fourth line: divercin V41 chromosomal locus (AJ224003). Genes *cbnB2*, *cbnBM1*, *cbnA*, *pisA* and *dvn41* encode the precursor bacteriocins (colored in black). Genes *cbiB2*, *cbiBM1*, *cbiA*, *pisI*, *dvnT2* and *dvnI* encode immunity proteins (colored in white). Genes *cbnT*, *cbaT*, *pisT* and *dvnT1* encode ABC transporter (italic hatching). Genes *cbnD*, *cbaC* and *pisE* encode transporter accessory protein (vertical hatching). The loci of carnobacteriocins B2, A and piscicolin 126 contain the *cbnS*–*cbnK*–*cbnR*, *cbaX*–*cbaK*–*cbaR* and *pisN*–*pisK*–*pisR* three-component regulatory system gene clusters, respectively (colored in gray). Only histidine protein kinase and response regulator protein encoded by *dvnK*–*dvnR* are found in the divercin locus (colored in gray).

The genes involved in carnobacterial bacteriocin production are generally clustered in operons. In the simplest case, corresponding to the sec-dependent divergicin A, the bacteriocin operon is composed of only the structural gene followed downstream by the gene encoding an immunity protein that protects the cell from is own bacteriocin (Worobo *et al*, 1995). Production of class IIa bacteriocins requires four genes as a minimum, including genes encoding bacteriocin, immunity protein, ABC of transport protein and its membrane-bound accessory protein. Genes for bacteriocin production may be encoded on the chromosome or on plasmids (Table 2). *Carnobacterium maltaromaticum* LV17 produces at least three class II bacteriocins. Carnobacteriocins A and B2 are encoded on different but compatible plasmids, pCP49 (72 kb) and pCP40 (61 kb), respectively. The carnobacteriocin BM1 structural gene and its immunity gene are localized on the chromosome. Activation and export of carnobacteriocin BM1 depend on genes located on plasmid pCP40 encoding carnobacteriocin B2 (Quadri *et al.*, 1997b; Rohde & Quadri, 2006). For carnobacteriocins BM1 and B2, a peptide-pheromone dependent quorum-sensing mode caused by the bacteriocin itself or an autoinducer peptide is involved in the regulation of bacteriocin production by two- and three-component signal transduction systems (Saucier *et al.*, 1995; Kleerebezem *et al.*, 2001; Nilsson *et al.*, 2002; Rohde & Quadri, 2006). The components of this regulatory system consist of an induction factor, a histidine protein kinase, and a response regulator. Four carnobacterial bacteriocin operons including genes for immunity proteins and regulatory proteins involved in bacteriocin expression and secretion have been sequenced (Fig. 1).

External parameters also affect bacteriocin production. Thus, increasing concentrations of NaCl (2–7%) reduced production of A9b/B2 bacteriocin (Himelbloom *et al.*, 2001; Nilsson *et al.*, 2002), increasing the temperature from below 19 to 25°C inhibited production of piscicolin 126 (Gursky *et al.*, 2006), and reducing the pH from 6.5 to 5.5 inhibited production of carnobacteriocins BM1 and B2 (Ahn & Stiles, 1990). Acetate (A9b bacteriocin) (Nilsson *et al.*, 2002) or the presence of a bacteriocin-sensitive strain belonging to the same genus (Sip *et al.*, 1998) induced bacteriocin production. It is clear that, in order to apply carnobacterial bacteriocins for the biopreservation of foods, detailed knowledge of the factors that influence production of the bacteriocin is necessary. Finally, it should be noted that the bacteriocinogenic activity may be lost, as shown for a *C. divergens* divercin V41-producing strain that retained only partial activity after being subjected to spray-drying (Silva *et al.*, 2002).

Interest in applying carnobacterial bacteriocins in foods and feeds has been directed towards inhibiting *Listeria monocytogenes* (Table 3) and spoilage microorganisms, to extend the shelf-life of lightly preserved seafood (Einarsson & Lauzon, 1995). These attempts have generally, but not always [e.g. regarding prevention of spoilage, Einarsson & Lauzon (1995) and Roller *et al.* (2002)], met with success, although diversity in sensitivity of the target organism has been observed (Brillet *et al.*, 2004). However, the occurrence of resistant *L. monocytogenes* target organisms has led to the suggestion that bacteriocin-negative LAB may be more suitable for practical use as bioprotective agents against *L. monocytogenes* in ready-to-eat foods (Nilsson *et al.*, 2004; Vermeiren *et al.*, 2006). Indeed, *L. monocytogenes* is inhibited by carnobacterial cultures that do not produce bacteriocins, and this is partly due to glucose depletion (Buchanan & Bagi, 1997; Nilsson *et al.*, 1999, 2005). One study also reported a successful application of cultures with no demonstrated bacteriocinogenic activity for extension of the shelf-life of vacuum-packed cold-smoked salmon (Leroi *et al.*, 1996). This effect was not, however, observed for a *C. divergens* strain that was unable to produce the class IIa divercin V41 (Richard *et al.*, 2003).

**Table 3:** Applications of antagonistic strains of *Carnobacterium* spp. against Listeria monocytogenes* in dairy, meat or fish food and feed products

Product (Species)	Mechanism	Reference
Dairy		
UHT milk (*Cm*)	Bacteriocin, other?	Buchanan & Klawitter (1992)
Whole milk (*Cm*)	Piscicolin 126	Wan *et al.* (1997)
Camembert cheese (*Cm*)	Piscicolin 126	Wan *et al.* (1997)
Fish and shellfish		
Cold-smoked salmon (*Cd*)	Divercin V41	Duffes *et al.* (1999a, b)
		Connil *et al.* (2002)
Cold-smoked salmon (*Cm*)	Piscocin V1, bacteriocin	Duffes *et al.* (1999a, b)
Cold-smoked salmon (*Cm*)	Carnobacteriocin B2, other	Nilsson *et al.* (1999, 2004)
Cold-smoked salmon (*Cm*)	Piscicocin CS526, other?	Yamazaki *et al.* (2003)
Pasteurized crabmeat (*Cm*)	Bacteriocin, other?	Buchanan & Klawitter (1992)
Meat		
Beef steaks (*Cm*)	Bacteriocinogenic substance	Schöbitz *et al.* (1999)
Canned dog food (*Cm*)	Bacteriocin, other?	Buchanan & Klawitter (1992)
Cooked chicken (*Cm*)	Bacteriocin, other	Campos *et al.* (1997)
Frankfurters (*Cm*)	Bacteriocin, other?	Buchanan & Klawitter (1992)
Ham paste (*Cm*)	Piscolin 126	Jack *et al.* (1996)
Ground meat (*Cm*)	Piscolin CS526 (freeze dried)	Azuma *et al.* (2007)
Sterile raw ground beef (*Cm*)	Bacteriocin, other?	Buchanan & Klawitter (1992)
Vegetables		
Canned creamed corn (*Cm*)	Bacteriocin, other?	Buchanan & Klawitter (1992)

* Studies using *Listeria innocua* as target organism e.g. Roller *et al.* (2002) have not been included in the table.

*Cd*, *Carnobacterium divergens*; *Cm, Carnobacterium maltaromaticum*.

Resistance of *L. monocytogenes* to the class IIa divergicin M35 was probably due to modification of the cell wall fatty acid composition (Naghmouchi *et al.*, 2006). *Listeria monocytogenes* strains resistant to the class IIa divercin V41 also showed substantial differences in protein expressions as compared with the wild type strain (Duffes *et al.*, 2000). A σ^54^-dependent PTS permease of the mannose family (EII_t_^Man^), which belongs to the phosphotransferase system (PTS), is responsible for sensitivity of *L. monocytogenes* to class IIa bacteriocins. These results suggested that EII_t_^Man^ encoded by the *mptACD* operon might be a target molecule for class IIa bacteriocins (Dalet *et al.*, 2001; Gravesen *et al.*, 2002). Recently, two genes coding for a glycerophosphoryl diester phosphodiesterase (*GlpQ*) and a protein with a putative phosphoesterase function (*Pde*) were identified as also being involved in the class IIa sensitivity of *En. faecalis* (Calvez *et al.*, 2007). Finally, it is important to note that the susceptibility of the target strain is affected by various environmental conditions such as NaCl and pH (Lebois *et al.*, 2004).

Another cause of concern when using carnobacteria for biopreservation is that *C. divergens* and *C. maltaromaticum* both produce tyramine, as discussed further below. Therefore, it has been suggested that a *C. divergens* strain in which the tyrosine decarboxylase gene is inactivated by mutagenesis could be used as a protective culture to prevent growth of *L. monocytogenes* in cold-smoked salmon (Brillet *et al.*, 2006). Currently, no carnobacterial culture, bacteriocin producing or not, is commercially applied for protection against the growth of *L. monocytogenes* or other bacterial pathogens or spoilage organisms in food.

### Effect of catabolic activities on sensory characteristics and safety of foods

As shown in Table 4, the catabolic activities of carnobacteria may result in sensory spoilage of inoculated fish and meat products. Whether this is so for cheese products is not clear. In naturally contaminated products, it seems other members of the bacterial community are typically more important with regard to sensory effects, including spoilage. It is of interest that spoilage was enhanced if moderate-spoilage strains of *C. maltaromaticum* were inoculated with nonspoilage *Vibrio* sp. strains or the moderate-spoilage organism *Brochothrix thermosphacta* into cold-smoked salmon that was subsequently vacuum-packaged (Joffraud *et al.*, 2006). However, this was not so for combinations of *C. maltaromaticum* and *Photobacterium phosphoreum* in this product. In addition, a spoilage synergy effect was observed for combinations of *B. thermosphacta* and *C. divergens*, *C. maltaromaticum* or *C. mobile* in MAP shrimp (Mejlholm *et al.*, 2005; Laursen *et al.*, 2006).

**Table 4:** Sensory effect of *Carnobacterium* spp. inoculated into dairy, fish or meat products

Food (storage conditions)	Species	Spoilage (sensory effect)	Important metabolites produced	Reference
Dairy				
Skimmed milk	*Cm*	Spoiled? (Malty aroma/flavor)	Branched alcohols and aldehydes*	Miller *et al.* (1974)
Fish and shellfish				
Cold-smoked salmon				
Vacuum, 4–8°C	*Cd*, *Cm*	Not or weakly spoiled (cheese/feet in some cases)	Not examined	Brillet *et al.* (2005)
Vacuum, 4–8°C	*Cd*, *Cm*	Not spoiled	Not examined	Duffes *et al.* (1999b)
MAP or vacuum, 5°C	*Cm*	Not spoiled	Not examined	Paludan-Müller *et al.* (1998)
Vacuum, 5°C	*Cm*	Not spoiled	Not examined	Nilsson *et al.* (1999)
Vacuum, 6°C	*Cm*	Not spoiled (butter, caramel, sour, fruity)	2,3-Butanedione, 2,3-pentanedione	Joffraud *et al.* (2001)
Vacuum, 6°C	*Cm*	Not spoiled [butter/plastic, rubbery and neutral (green/cooked meat)]	Not examined	Stohr *et al.* (2001)
Vacuum, 8°C	*Cm*	Lightly spoiled (grassy, fruity notes)	Not examined	Joffraud *et al.* (2006)
Shrimp (MAP, 5°C)	*Cd*, *Cm*	Spoiled (chlorine, chemical, malty, nutty, sour)	Ornithine, ammonia, acetic acid, alcohols, aldehydes, ketones, 2,4,6-trimethylpyridine	Mejlholm *et al.* (2005)Laursen *et al.* (2006)
	*Cm* (L)†	Not spoiled (grass/hay, weak chlorine)	Ornithine, ammonia, acetic acid, alcohols, aldehydes, ketones	Laursen *et al.* (2006)
	*Cmo*	Not spoiled (yogurt-like)	Organic acids, alcohols, ketones	Laursen *et al.* (2006)
Meat				
Beef				
Vacuum, 2°C	*Cm*	Not spoiled/spoiled‡	Not examined	Leisner *et al.* (1995)
Vacuum, 4°C, normal pH	*Cd*	Not indicated (butter, acid)	Not examined	Borch *et al.* (1996)
Vacuum, 4°C, high pH	*Cd*	Not indicated (acid, slightly sulfurous)	Not examined	Borch *et al.* (1996)
In air, 7°C	*Cm*	Spoiled	Not examined	Leisner *et al.* (1995)
Cured bologna (4 or 9°C)	*Cv*	Green discoloration	Presumably by H_2_O_2_	Peirson *et al.* (2003)
Ham, cooked, sliced (vacuum, 5 or 7°C)	*Cm*	Spoiled	Branched alcohols and aldehydes	Budde *et al.* (2003)
Cooked, minced sausage (24–15°C)	*Cm*	Sausage/fatty odor	α-Ketoisocaproic acid, hydroxy-α-ketoisocaproic acid, 3-methylbutanoic acid	Larrouture-Thiveyrat *et al.* (2003)

* 2-Methylpropanal, 2-methylpropanol, 3-methylbutanal, 3-methylbutanol (both ham and milk) and 2-methylbutanal (ham). For milk, the production of these compounds was determined in a laboratory medium and not milk *per se*.

† Phenotypical cluster L (Laursen *et al.*, 2005).

‡ Spoilage observed at an initial density of log 4 CFU cm^−2^ but not at log 2 CFU cm^−2^.

*Cd*, *Carnobacterium divergens*; *Cm*, *Carnobacterium maltaromaticum*; *Cmo, Carnobacterium mobile*; *Cv*, *Carnobacterium viridans*.

We will first focus on catabolic reactions with carbohydrates and/or organic acids as substrates and with pyruvic acid as an intermediate metabolite. Respiration might occur in the presence of hematin, as shown for *C. maltaromaticum* (Meisel *et al.*, 1994), and, indeed, this species consumes substantial proportions of oxygen during exponential growth under aerobic conditions (Borch & Molin, 1989). *Carnobacterium* spp. are, however, considered to be homofermentative organisms that produce lactic acid from glucose. The presence of the glycolytic pathway in *C. divergens* has been demonstrated (de Bruyn *et al.*, 1987, 1988). It can be debated whether carnobacteria should instead be considered facultative or atypical heterofermentative organisms, as *C. divergens* and *C. maltaromaticum* are able to utilize ribose and gluconic acid as substrates for growth, and may produce acetic acid, formic acid, and CO_2_ (e.g. Shaw & Harding, 1984; de Bruyn *et al.*, 1988; Borch & Molin, 1989; Leisner, 1992) as end-products of some secondary decarboxylation/dissimilation reactions of pyruvic acid (de Bruyn *et al.*, 1988; Schillinger & Holzapfel, 1995). Indeed, carnobacteria were initially described as heterofermenters (Collins *et al.*, 1987). Acetic acid production by *C. divergens* and *C. maltaromaticum* can be substantial during growth in laboratory media under aerobic conditions or in MAP shrimp, and quantitatively it can exceed lactic acid production (Borch & Molin, 1989; Laursen *et al.*, 2006). Production of acetic acid by *C. maltaromaticum* is also increased relative to lactic acid if glucose is substituted by ribose (Leisner, 1992). Furthermore, *C. maltaromaticum* can produce large amounts of ethanol from glucose and ribose during growth in a shrimp extract under anaerobic conditions (Leisner, 1992).

Acetoin can be generated by *C. maltaromaticum* from pyruvic acid (Borch & Molin, 1989), and this reaction is also found for *C. divergens* and *C. gallinarum* but not for *C. alterfunditum*, *C. funditum*, *C. inhibens* and *C. viridance*, according to reactions in the Voges–Proskauer test (Collins *et al.*, 2002; Holley *et al.*, 2002). *Carnobacterium mobile* gives variable reactions, and information is not available for *C. pleistocenium* (Collins *et al.*, 1987; Pikuta *et al.*, 2005). The factors affecting acetoin production are not well known, but production is increased by resting cells of *C. maltaromaticum* in the presence of hematin (Meisel *et al.*, 1994). In addition, two out of four strains of *C. divergens* and *C. maltaromaticum* isolated from mozzarella cheese were reported to be able to metabolize citric acid, but the potential sensory role of this reaction, e.g. by production of acetoin and diacetyl, was not examined (Morea *et al.*, 1999). Production of diacetyl and 2,3-pentanedione by *C. maltaromaticum* during growth in cold-smoked salmon resulted in a butter-like odor but not in spoilage (Joffraud *et al.*, 2001) (Table 4). In conclusion, even, if carbohydrate catabolism by carnobacteria appears to result in a diverse number of metabolites, these have generally a limited effect on the sensory attributes of foods. H_2_O_2_ may be produced by *C. divergens* (Borch & Molin, 1989), and formation of this compound by *C. viridans* has been associated with spoilage in the form of green discoloration of cured Bologna ham (Table 4).

Whether carnobacteria possess proteolytic activities of potential importance for taste in products such as cheese is not known, and this deserves further investigation. In contrast, metabolites resulting from the degradation of amino acids certainly cause sensory effects in foods (Table 4). Generation of branched alcohols and aldehydes (2-methyl-1-butanal, 2-methyl-1-butanol, 3-methyl-1-butanal, 3-methyl-1-butanol, 2-methylpropanal, and 2-methylpropanol) by transamination, decarboxylation and reduction of the amino acids valine, leucine and isoleucine appears to be particularly important. Production of some of these compounds, such as 3-methyl-1-butanal, is strain or species dependent (Larrouture *et al.*, 2000). Production by a *C. maltaromaticum* strain of 3-methylbutanal, 3-methylbutanol and 3-methylbutanoic acid from leucine was, in general, increased at pH values of 6.5 or higher and in the presence of increased concentrations of α-ketoisocaproic acid and glucose (Larrouture-Thiveyrat & Montel, 2003). This strain affected the odor and reduced the leucine content of a sausage mince, but a clear causal relationship was not demonstrated (Larrouture-Thiveyrat *et al.*, 2003). Production by *C. maltaromaticum* of alcohols and aldehydes from valine, leucine and/or isoleucine resulted in a malty, green aroma in skimmed milk and shrimp, and has also caused spoilage of cured ham (Table 4). The association between the presence of these compounds and spoilage is, however, not always straightforward, as production of 3-methyl-1-butanal and/or 3-methyl-1-butanol in shrimp by certain *C. maltaromaticum* strains did not result in malty off-flavors (Laursen *et al.*, 2006).

Production of NH_4_^+^ from arginine as a result of its catabolism catalyzed by the arginine deaminase pathway (Leisner *et al.*, 1994b) may, in theory, cause spoilage of shrimp or squid that contain high levels of free arginine (Asakawa *et al.*, 1981; Hirano *et al.*, 1992). *Carnobacterium maltaromaticum* and *C. divergens* but not *C. mobile* were able to produce NH_4_^+^ in MAP shrimp, but this was not the cause of spoilage of this product (Laursen *et al.*, 2006).

Production of indole from the amino acid tryptophan is also a potential cause of spoilage, although *C. divergens*, *C. maltaromaticum* and *C. mobile* are all unable to carry out this reaction in standard laboratory media (Laursen *et al.*, 2006). It has, however, been observed that *C. divergens* and strains belonging to a major phenotypic cluster of *C. maltaromaticum* were able to use tryptophan as a substrate during growth in MAP shrimp (Laursen *et al.*, 2006). Whether this resulted in generation of indole was not examined.

Production of tyramine from tyrosine is the only known metabolic reaction by *Carnobacterium* spp. that constitutes a cause of concern regarding food safety. Not all carnobacterial species possess this ability, as *C. mobile* does not produce tyramine during growth in shrimp, and variation exists among different strains and phenotypic clusters of *C. divergens* and *C. maltaromaticum* for amounts of tyramine produced (Leisner *et al.*, 1994a; Masson *et al.*, 1996; Bover-Cid & Holzapfel, 1999; Laursen *et al.*, 2006). Tyramine production by one *C. divergens* strain was at its maximum during the stationary growth phase and at a low initial pH in the presence of 0.6% glucose, whereas NaCl (10%) was inhibitory (Masson *et al.*, 1997). Regardless of strain variation and the effects of environmental parameters, tyramine production by *C. divergens* and/or *C. maltaromaticum* has been reported in a range of foods, including meat (up to 28 mg kg^−1^) (Edwards *et al.*, 1987), a meat–fat mixture (up to 121 mg kg^−1^) (Masson *et al.*, 1999), cold-smoked salmon (up to *c*. 370 mg kg^−1^) (Duffes *et al.*, 1999b; Jørgensen *et al.*, 2000; Connil *et al.*, 2002; Brillet *et al.*, 2005), frozen and thawed salmon (up to 40–60 mg kg^−1^) (Emborg *et al.*, 2002), and shrimp (up to 20–60 mg kg^−1^) (Laursen *et al.*, 2006). These levels have no adverse effects on most consumers, but for sensitive individuals, e.g. with reduced monoamine oxidase activity due to medication or hereditary deficiency, very little tyramine can cause migraine headaches, and an intake of no more than 5 mg of tyramine per meal has been recommended (McCabe, 1986). Typical consumption of fish and meat products is 50–150 g per meal. As described above, *C. divergens* and/or *C. maltaromaticum* are able to form *c*. 20–370 mg tyramine kg^−1^ in these products, corresponding to *c*. 1–55 mg of tyramine per meal. Consequently, tyramine formation by carnobacteria in specific foods can represent a hazard for sensitive individuals who might suffer migraine headaches. This risk for sensitive individuals can be minimized by restricting their consumption of fish and meat to products that are newly processed, i.e. that are not close to the declared shelf-life expiry day.

Growth of *Carnobacterium* spp. in food products may result in accumulation of a range of volatile alcohols, ketones, hydrocarbons, and other compounds (Laursen *et al.*, 2006). The metabolic reactions leading to these products have not been studied in detail for *Carnobacterium*, in contrast to the situation for other LAB (Smit *et al.*, 2005), but may be a result of NAD+-generating reactions or nonenzymatic reactions. Several ketones have characteristic odors, but whether they contribute to spoilage off-flavors as a result of carnobacterial metabolic activity is not well understood. With regard to lipids, *C. maltaromaticum* has been reported to hydrolyze tributyrin, a triglyceride with butyrate as fatty acid (Papon & Talon, 1988), but it is not known whether this lipase activity contributes to flavor changes of dairy, fish and meat products. *Carnobacterium divergens* and *C. maltaromaticum* were not able to limit oxidation of linoleic acid during growth (Talon *et al.*, 2000). This indicates that these species may not prevent quality deterioration of meat products by lipid oxidation. Finally, *C. maltaromaticum* has been shown to cause a softer texture of salmon fillets when inoculated in high numbers (Morzel *et al.*, 1997).

It is worth noting that interspecies and intraspecies differences exist regarding carnobacterial spoilage capacity. Laursen *et al.* (2006) showed that *C. mobile* and a minor phenotypic cluster of *C. maltaromaticum* did not spoil MAP shrimp, in contrast to what was seen with *C. divergens* and the major phenotypic cluster of *C. maltaromaticum*. It would be of interest to examine whether similar heterogeneities exist for *Carnobacterium* spp. growing in other dairy, fish and meat products. As environmental parameters readily affect the metabolism of carbohydrates and amino acids, it is not surprising that product-specific and storage-specific conditions also play important roles. The ability of two *C. maltaromaticum* strains to relatively rapidly spoil meat during growth under aerobic conditions at 7°C but not or only after a long storage period in vacuum packs at 2°C serves as an example (Table 4) (Leisner *et al.*, 1995).

## Carnobacteria as pathogenic organisms and/or probiotic cultures

Two human clinical cases caused by opportunistic infection with *C. maltaromaticum* and a *Carnobacterium* sp. have been described (Xu *et al.*, 1997; Chmelar *et al.*, 2002). Two clinical isolates of *C. divergens* have also been obtained (accession number AY650920). Carnobacteria are not known members of the human gastrointestinal microbial community, unlike several other LAB. The exceptions are an unpublished study reporting an uncultured *Carnobacterium* sp. clone from cow rumen (AY244976) and two studies demonstrating the presence of carnobacteria in pig and horse effluent-impacted environments (DQ337521; DQ337531) (Simpson *et al.*, 2004). Nevertheless, it is safe to conclude that the presence of *Carnobacterium* spp. in food does not present a risk factor for human illness, except for the production of the biogenic amine, tyramine, as discussed previously. Neither do *Carnobacterium* spp. appear to present a risk for nocosomial infections in hospitals and similar institutions, although they have, in one instance, been isolated from contaminated blood plasma (Thornley & Sharpe, 1959).

The presence of virulence factors in carnobacteria is not well documented. Thus *C. maltaromaticum* strains isolated from diseased fish lacked hemolytic and phospholipolytic activities (Hammes & Hertel, 2003). *Carnobacterium viridans* shows, however, β-hemolytic activity on sheep blood agar (Holley *et al.*, 2002). Although several isolates of carnobacteria produce bacteriocins, none of these compounds has been reported to exert a cytolytic activity as shown for the *En. faecalis* class I-related bacteriocin cytolysin (Gilmore *et al.*, 1994). Regarding chemotherapeutic agents, fish pathogenic strains of *C. maltaromaticum* were resistant to several of the agents widely used in aquaculture, such as oxytetracycline, quinolones, nitrofurans and potentiated sulfonamides, but sensitive to erythromycin (Michel *et al.*, 1986; Baya *et al.*, 1991; Toranzo *et al.*, 1993a). This species is not resistant to lysozyme from salmonid eggs, which supports the idea that it is not vertically transmitted, at least in this fish species (Yousif *et al.*, 1994). Finally, it should be noted that two clinical isolates of *C. divergens* have been reported to possess a gene encoding a new class of penicillinase (AY650920). It will be of interest to further explore the distribution of this enzyme among carnobacterial species as well as its functionality.

It has been documented that some strains of *C. maltaromaticum* are pathogenic for several fish species, including Australian salmonids (Humphrey *et al.*, 1987), carp (Michel *et al.*, 1986), rainbow trout (Hiu *et al.*, 1984; Baya *et al.*, 1991; Starliper *et al.*, 1992; Toranzo *et al.*, 1993a, b), striped bass and channel catfish (Baya *et al.*, 1991; Toranzo *et al.*, 1993b), and salmon (Hiu *et al.*, 1984; Michel *et al.*, 1986). Pathologic effects vary, and include, for example, septicemia, peritonitis, exophthalmia, accumulation of ascitic fluid, and hemorrhages. In most (but not all) instances, the virulence appears, however, to be low and only causes problems in fish undergoing severe stress, e.g. due to spawning (Michel *et al.*, 1986; Starliper *et al.*, 1992). It is, therefore, not surprising that *C. maltaromaticum* and *C. maltaromaticum*-like strains are also found in the intestine or gills of healthy fish, including Arctic charr (Ringø*et al.*, 1998, 2001; Ringø & Olsen, 1999), Atlantic cod (Seppola *et al.*, 2005; Ringø*et al.*, 2006), Atlantic salmon (Ringø & Holzapfel, 2000; Ringø*et al.*, 2000, 2001), brown bullhead (Baya *et al.*, 1991), trout (González *et al.*, 1999; Spanggaard *et al.*, 2001; Pond *et al.*, 2006), and various freshwater fish (González *et al.*, 1999, 2000). Ringø*et al.* (2005) also summarize some of these findings.

The presence of other carnobacterial species in healthy fish has also been reported, including *C. divergens* [Arctic charr (Ringø & Olsen, 1999; Ringø*et al.*, 2002a), Atlantic salmon, Atlantic cod and wolffish (Ringø*et al.*, 2001), trout (Spanggaard *et al.*, 2001; Kim & Austin, 2006b), and freshwater fish (González *et al.*, 2000)], *C. alterfunditum*-like [rainbow trout (Spanggaard *et al.*, 2001)], *C. funditum*-like [Arctic charr and Atlantic cod (Ringø*et al.*, 2002b, 2006)], *C. gallinarum* [Atlantic cod (Seppola *et al.*, 2005)], *C. inhibens* [Atlantic salmon (Jöborn *et al.*, 1999)], *C. mobile*-like [Arctic charr and Atlantic cod (Ringø & Olsen, 1999; Ringø*et al.*, 2006)], and *Carnobacterium* spp. [Arctic charr (Ringø*et al.*, 2001) and carp (Hagi *et al.*, 2004)]. Clearly, further research is necessary to clarify under what circumstances *Carnobacterium* spp. may be pathogenic for fish and whether the infective capability is species and clone specific.

The reported pathogenicity of *C. maltaromaticum* has not hindered research into the possibility of using other carnobacteria as probiotic cultures in aquaculture, including *Carnobacterium* sp. (Irianto & Austin, 2002), *C. alterfunditum* (Spanggaard *et al.*, 2001), *C. divergens* (Gildberg *et al.*, 1995, 1997; Spanggaard *et al.*, 2001), and *C. inhibens* (Jöborn *et al.*, 1997). Isolates of these species, in addition to *C. maltaromaticum*, exhibit inhibitory activity towards bacterial fish pathogens (Ringø & Holzapfel, 2000; Gram & Ringø, 2005; Ringø*et al.*, 2005). *Carnobacterium divergens* and *C. maltaromaticum* enhance the cellular and humoral immune responses and cytokine expression ratios of rainbow trout (Kim & Austin, 2006a, b). Use of carnobacteria as probiotics leads to increased survival of the fish in some instances [larvae of cod fry and Atlantic salmon fry (Gildberg *et al.*, 1995, 1997), rainbow trout (Irianto & Austin, 2002), and salmon (Robertson *et al.*, 2000)], but not in others [larvae of cod fry (Gildberg & Mikkelsen, 1998), and rainbow trout (Spanggaard *et al.*, 2001)]. One study showed that *in vitro* exposure to *C. divergens* did not reverse the effects of bacterial pathogens, although these were alleviated (Ringø*et al.*, 2007). Additional research is necessary to validate the usefulness of carnobacteria as probiotic cultures.

*Carnobacterium maltaromaticum* has also been reported to be pathogenic for the fruit fly, *Drosophila melanogaster*, upon injection into the thorax (Jensen *et al.*, 2007). This specific case of pathogenesis is probably best understood as a result of opportunistic infection, although *C. maltaromaticum* (but not *C. divergens*, *C. gallinarum*, *C. inhibens*, *C. mobile* or *C. viridans*) exerts chitinolytic acitivity (Leisner & Ingmer, unpublished data), a feature that can be perceived as targeting the chitin-containing exoskeleton of insects. Indeed, insects may serve as a host for this species (Shannon *et al.*, 2001).

## Genomics

The complete chromosomes of many LAB species have now been sequenced. Currently, 19 complete genome sequences of streptococci and 18 complete genome sequences of the nonpathogenic LAB representing 14 species from the order *Lactobacillales* are available (Makarova & Koonin, 2007). However, no genome sequence or physical genetic map is available for *Carnobacterium*. The LAB have relatively small genomes for nonobligatory bacterial parasites or symbionts, ranging from *c*. 1.6 to *c*. 3 Mb. For *C. divergens* and *C. pleistocenium*, the genome size was estimated to 3.2 Mb, and for *C. alterfunditum* pf4^T^, it was estimated to be 2.9 Mb (Daniel, 1995; Pikuta *et al.*, 2005). At the time of writing (March 2007), there is only one *Carnobacterium* genome sequencing project. *Carnobacterium* sp. AT7, a piezophilic strain isolated from the Aleutian trench at a depth of 2500 m, is being sequenced for the Moore Foundation Marine Microbial Genome Sequencing Project with a grant to the J. Craig Venter Institute. The data already available indicate that the *Carnobacterium* sp. AT7 genome contains 2.4 Mb and encodes 2388 proteins (http://www.genomesonline.org) (Liolios *et al.*, 2006). *Carnobacterium* sp. AT7 is closely related to *C. alterfunditum* and *C. pleistocenium* (Lauro *et al.*, 2007).

To date, knowledge on the genes and DNA sequences of *Carnobacterium* is comparatively sparse, concerning mostly bacteriocin-related genes in the species *C. divergens* and *C. maltaromaticum* (Table 2) and 16S rRNA and 16S–23S rRNA gene intergenic spacer sequences (Kabadjova *et al.*, 2002; Rachman *et al.*, 2004). Genes involved in some important carnobacterial metabolic traits have been sequenced (Table 5). The genetic information obtained is, for most sequences, derived from just one strain, and may therefore be strain specific, as shown for a number of *L. monocytogenes* genes (Nelson *et al.*, 2004).

**Table 5:** Summary of known genes and DNA sequences in Carnobacterium*

Function	Species/Strain	Gene	Accession number	References†
Bacteriocin immunity				
To carnobacteriocin A	*Cm*/LV17A	*cbiA*‡	AF207838	Franz *et al.* (2000)
To carnobacteriocin BM1	*Cm*/LV17B	*cbiBM1*	L29058	Quadri *et al.* (1994)
To carnobacteriocin B2	*Cm*/LV17B, CP52	*cbiB2*‡	L47121	Quadri *et al.* (1995)
				Herbin *et al.* (1997)
To divercin V41	*Cd*/V41	*dvnT2*, *dvnI*	AJ224003	Métivier *et al.* (1998)
To divergicin A	*Cd*/LV13	*dviA*‡	DQ087597	Worobo *et al.* (1995)
To piscicolin 126	*Cm*/JG126	*pisI*	AF275938	Gibbs *et al.* (2000) (U)†
Regulation of bacteriocin expression/secretion				
Carnobacteriocin A	*Cm*/LV17A	*cbaX*, *K*, *R*‡	AF207838	Franz *et al.* (2000)
Carnobacteriocin BM1 andB2: three-component regulatory system	*Cm*/LV17B	*cbnS*, *K*,*R*‡	L47121	Quadri *et al.* (1997b)
				Kleerebezem *et al.* (2001)
				Rohde & Quadri (2006)
Divercin regulatory system	*Cd*/V41	*dvnK*, *R*	AJ224003	Métivier *et al.* (1998)
Piscicolin 126 regulatory system	*Cm*/JG126	*pisN*, *K*, *R*	AF275938	Gibbs *et al.* (2000) (U)
Bacteriocin ABC transporter				
Carnobacteriocin A	*Cm*/LV17A	*cbaT*,*C*‡	AF207838	Franz *et al.* (2000)
Carnobacteriocin BM1 and B2	*Cm*/LV17B	*cbnT*,*D*‡	L47121, L29058	Quadri *et al.* (1997)
				Kleerebezem *et al.* (2001)
				Rohde & Quadri (2006)
Divercin	*Cd*/V41	*dvnT1*	AJ224003	Métivier *et al.* (1998)
Piscicolin 126	*Cm*/JG126	*pisT*, *E*	AF275938	Gibbs *et al.* (2000) (U)
Metabolism				
Alanine dehydrogenase	*Csp*/st2	*ald*	AF070714	Galkin *et al.* (1999)
ATP synthase α-subunit	*Cd*/LMG 9199^T^	*atpA* partial	AJ843296	Naser *et al.* (2005)
	*Cm*/LMG 9839^T^	*atpA* partial	AJ843298	Naser *et al.* (2005)
Glutamate racemase	*Csp*/st2	*Glr*	AF263927	Galkin *et al.* (2000) (U)
Glycosyl hydrolases	*Cm*/BA	*agaA*	AF376480	Coombs & Brenchley (2001)
		*bgaC*	AF376481	Coombs & Brenchley (2001)
		*bgaB*	AF184246	Coombs & Brenchley (1999)
Homoserine dehydrogenase and aromatic amino acid aminotransferase	*Cm*/545	*hdhCP*, *araTCP*	AY029372	Larrouture-Thiveyrat *et al.* (2001) (U)
Penicillinase	*Cd*/BM4489	*cad-1*	AY650920	Perichon *et al.* (2004) (U)
Phenylalanyl-tRNA synthase α-subunit	*Cm*/LMG 6903^T^	*pheS*	AM168425	Naser *et al.* (2006)
Superoxide dismutase	*Cm*/3364-01	*sodA*, partial	AM490329	Michel *et al.* (2007)
	*Cm*/NCIMB 2264	*sodA*, partial	AM490310	Michel *et al.* (2007)
	*Ci*/CIP 106863^T^	*sodA*, partial	AM490313	Michel *et al.* (2007)
Tyrosine decarboxylase	*Cd*/V41	*tdc*	DQ336701	Brillet *et al.* (2005) (U)
				Coton *et al.* (2004)
Miscellaneous				
Recombinase A	*Cm*/LMG 6903^T^	*recA*, partial	AJ621690	Felis *et al.* (2005) (U)
RNAse RH	*Csp*/st2	*rph*, partial	AF263927	Galkin *et al.* (2000) (U)
Theta-type plasmid	*Cd*/LV13	*pCD3.4*‡	DQ087597	Van Belkum & Stiles (2006)

* For bacteriocin structural genes, see Table 2.

† U, unpublished sequence (National Center for Biotechnology Information Genome Library, http://www.ncbi.nlm.nih.gov).

‡ The sequences are encoded by plasmids.

*Cd, Carnobacterium divergens*; *Ci*, *Carnobacterium inhibens*; *Cm*, *Carnobacterium maltaromaticum*; *Csp*, *Carnobacterium* sp.

Clearly, our knowledge of important phenotypes of strains and species of *Carnobacterium* would benefit from the availability of a complete genome sequence for a strain representative of *C. maltaromaticum*, which is the species with most importance for the food and aquaculture industries. The most extensive study on phenotypic variation of *C. maltaromaticum* revealed that the majority of strains studied belonged to one phenotypic cluster (cluster H) (Laursen *et al.*, 2005). Thus, in our opinion, a suitable candidate for a whole genomic sequence could be either *C. maltaromaticum* LMG 22901 (isolated from pork meat), LMG 22899 (isolated from cod) or LMG 22898 (isolated from salmon), which all belong to this phenotypic cluster. LMG 22901 does not harbor plasmids (Laursen, unpublished), and therefore appears to be a prime candidate for such an endeavor.

## Concluding remarks

We have come some way towards understanding important aspects of the presence of carnobacteria in foods and the environment, particularly concerning the genetics of carnobacterial bacteriocins and their application. However, there are important areas where knowledge on these bacteria is limited. These include the following:

1. Distribution and quantitative microbial ecology: *Carnobacterium* spp. have not been reported from a number of habitats, such as plants, fermented vegetable foods and the gastrointestinal system of birds and mammals, including humans, that have otherwise been associated with several genera and species of LAB. This may, in some instances, be due to faulty methodology for detecting carnobacteria. Generally, however, our quantitative understanding is limited regarding factors that influence inactivation, survival or growth of carnobacteria in various natural environments. In foods, more detailed information is available, but mathematical models for quantitative prediction of processing, product and storage effects on growth and metabolic activity, including bacteriocin and tyramine formation, awaits further development together with recording of responses in databases. In addition, further studies are needed to elucidate mechanisms underlying the relationship between environmental parameters and kinetic responses of carnobacteria.
2. Routes of food contamination: To reduce the negative effects of carnobacteria in foods (spoilage metabolites and tyramine), further information on routes of contamination is desirable. In fact, very little research has been performed so far concerning this aspect of carnobacteria in food.
3. Metabolic activity: It is known that carnobacteria, or at least *C. maltaromaticum* and *C. divergens*, have the capacity to produce a wide range of metabolites in chilled food. Further studies on the importance of these metabolites with respect to positive and negative sensory attributes of various foods are, however, needed.
4. Diversity: We are beginning to appreciate how interspecies and intraspecies variation determine the positive and negative effects of the presence of carnobacteria in food and the environment, but we still do not understand how such variation affects, for instance, their survival in food-processing plants and contamination of foods. The significance of such variation for their potential as fish pathogens also needs to be substantiated. This is also the case for their role as spoilage organisms in meat and fish products, with the exception of a few products such as vacuum-packed smoked salmon and cooked MAP shrimps.
5. Genomics: Representative genome sequences would offer a very valuable road map in order to answer many of the outstanding questions associated with this important genus.

## Statement

Reuse of this article is permitted in accordance with the Creative Commons Deed, Attribution 2.5, which does not permit commercial exploitation.
